# The effect of fibroblast growth factor 7 on human dental pulp stem cells for differentiation to AQP5‐positive and αSMA‐positive cells *in vitro* and *in vivo*

**DOI:** 10.1002/cre2.423

**Published:** 2021-03-30

**Authors:** Yoshihiko Akashi, Atsushi Nemoto, Kei Nakajima, Katsutoshi Kokubun, Satoshi Murakami, Takashi Inoue, Kenichi Matsuzaka

**Affiliations:** ^1^ Department of Pathology Tokyo Dental College Tokyo Japan; ^2^ Department of Oral Pathology Matsumoto Dental University Shiojiri Japan; ^3^ Tokyo Dental College Tokyo Japan

**Keywords:** regeneration, salivary glands, stem cell(s), wound healing

## Abstract

**Objectives:**

Transplantation of stem cells into wounds has become popular in regeneration therapies. As stem cells for transplantation, human dental pulp stem cells (hDPSCs) are known to be pluripotent cells that are relatively easy to collect from the pulp of deciduous or wisdom teeth. The purpose of this study was to investigate whether hDPSCs treated with fibroblast growth factor 7 (FGF7) would contribute to the regeneration of wounded rat submandibular glands (SMGs).

**Materials and methods:**

In *in vitro* studies, hDPSCs were treated with or without FGF7 and mRNA expression levels were examined at days 3, 7 and 14 using qRT‐PCR. The target genes analyzed were *BMI1* as an undifferentiated marker, *AQP5* as an acinar cell marker, *CK19* as a ductal epithelial cell marker, *αSMA* as a myoepithelial cell marker and *VIMENTIN* as a fibroblast marker. In *in vivo* studies, hDPSCs treated with or without FGF7 for 14 days were mixed with type I collagen gels and were transplanted into wounded rat SMGs. Hematoxylin–Eosin and immunohistochemical staining were performed at days 3 and 7, and the numbers of positive cells were counted. The primary antibodies used were against BMI1, AQP5, αSMA, PanCK and VIMENTIN.

**Results:**

In the *in vitro* studies*,* mRNA levels of *BMI1* were decreased and *αSMA* were increased at days 3, 7 and 14, while *AQP5* was increased at day 14 in the FGF7 group. In the *in vivo* studies, the proliferation of hDPSCs and cell islands was observed at day 7 in the FGF7 group. Few BMI1‐positive cells were observed, while numbers of AQP5‐positive and αSMA‐positive cells were increased at days 3 and 7 in the FGF7 group. Moreover, cell islands were AQP5‐positive.

**Conclusion:**

These results suggest that FGF7‐treated hDPSCs differentiate into AQP5‐positive and αSMA‐positive cells. Moreover, AQP5‐positive cell aggregations were formed.

## 1 INTRODUCTION

The secretion of saliva is related to swallowing, pronunciation, lubrication, antibacterial action, buffering action, taste, and so on (Carlson & Ord, 2008; Vissink, Burlage, Spipijkervet, Jansma, & Coppes, 2003; Vissink, Jansma, Spijkervet, Burlage, & Coppes, 2003). Salivary glands that secrete saliva are tissues that are relatively susceptible to damage from trauma, injury, radiation, drug injury, Sjögren syndrome and so on, and such damage often causes a decrease in salivary secretion, which can significantly reduce the patient's quality of life (Atkinson, Grisius, & Massey, 2005; Fox, 1998; Ship, Pillemer, & Baum, 2002). Generally, salivary glands that are already functionally differentiated are classified as tissues with low regenerative capacity. However, currently, it has been reported that acinar cells regenerate salivary glands by self‐renewal (Aure, Konieczny, & Ovitt, 2015) and it has also been reported that stem/progenitor cells present in ductal cells contribute only to ductal cells, not to acinar cells (Kwak, Alston, & Ghazizadeh, 2016). Moreover, there is also a report of salivary gland regeneration using stem cells (Tanaka et al., 2018) and research in this area is being actively conducted. Therefore, research studies on the regeneration of damaged salivary glands are very important and informative.

In the case of salivary gland wound healing, non‐specific granulation tissue, which consists of capillaries and fibroblasts, can appear and eventually forms scar tissue that can disrupt salivary gland function (Man, Ball, Marchetti, & Hand, 2001; Takahashi, Schoch, & Walker, 1998). To avoid scar tissue formation in wounded salivary glands, granulation tissue from surrounding areas must be excluded using barrier membrane techniques and stem cells need to be induced in the wound areas for regeneration. However, only a few stem cells are present in salivary glands as noted above. Therefore, tissue engineering techniques in which three elements, cells, scaffolds and growth factors, are required for the regeneration of salivary glands.

It has been reported that regenerative treatments include the transplantation of stem cells into wounds, and mesenchymal stem cells represented by adipose stem cells and bone marrow stem cells are used for salivary gland regeneration (Elsaadany, Zakaria, & Mousa, 2019; Kim et al., 2019). However, human dental pulp stem cells (hDPSCs) are known to be pluripotent cells that can be collected relatively easily from deciduous teeth and from wisdom teeth, but there are no reports of using them for transplantation. We hypothesized that the use of hDPSCs as stem cells would be an effective approach for salivary gland regeneration following salivary gland injury.

There have been many reports that type I collagen gels play important roles in cellular proliferation and differentiation *in vitro* (Auger et al., 1998; Gospodarowicz, Greenburg, & Birdwell, 1978; Lombaert et al., 2008; Murray, Stingl, Kleinman, Martin, & Katz, 1979; Parry & Creamer, 1980; Sattler, Michalopoulos, Sattler, & Pitot, 1978; Wicha, Liotta, Garbisa, & Kidwell, 1979) and *in vivo* (Nanduri et al., 2014; Yang et al., 2014). It has also been reported that various types of cells, including stem cells, can easily migrate into type I collagen gels (Kobayashi, Matsuzaka, & Inoue, 2016; Kobayashi, et al., Kobayashi et al., 2019). Kobayashi et al. (2016) reported that stem cells migrated into type I collagen gels containing bFGF in wounded salivary glands. Further, they reported that specific proteins that are related to salivary gland components were expressed but the glands did not regenerate structurally, and they concluded that other growth factor(s) might be necessary to stimulate structural regeneration.

It is known that various growth factors, such as fibroblast growth factor (FGF), transforming growth factor (TGF), epidermal growth factor (EGF), insulin‐like growth factor (IGF), platelet‐derived growth factor (PDGF), hepatocyte growth factor (HGF) and bone morphogenic protein (BMP), are necessary for tissue regeneration and wound healing. Fibroblast growth factor 7 (FGF7) is known as a keratinocyte growth factor that is important in epithelial regeneration and differentiation (Kera, Yuki, & Nogawa, 2014; Morita & Nogawa, 1999).

In this study, the effects and behavior of transplanting type I collagen gels containing hDPSCs treated with FGF7 into wounded rat SMGs was investigated *in vitro* and *in vivo*.

## 2 MATERIALS AND METHODS

### 2.1 Cells

hDPSCs (PT‐5025; Lonza, Basel, Switzerland) derived from adult human third molars were purchased from Lonza for use in this study. Those cells are primary cultures that express CD105, CD166, CD29, CD90 and CD73, but are negative for CD133, CD34 and CD45. Those hDPSCs have the phenotypic and functional characteristics of bone marrow‐derived mesenchymal stem cells.

### 2.2 Cell culture medium

The dental pulp stem cell growth medium (DPSC‐GM) was prepared by adding the contents of a DPSC SingleQuots Kit (PT‐4516; Lonza, Basel, Switzerland) to DPSC Basal Medium (PT‐3927; Lonza, Basel, Switzerland). The DPSC SingleQuots Kit contains human Dental Pulp Stem Cell Growth Supplement, L‐glutamine, ascorbic acid and gentamicin/amphotericin‐B.

### 2.3 Preparation of FGF7 in DPSC‐GM

Recombinant FGF7 powder (10 μg) (FUJIFILM Wako Pure Chemical Corporation, Osaka, Japan) was dissolved in 1.0 mL phosphate buffered saline (PBS) to produce the FGF7 stock solution (10 μg/mL). This FGF7 stock solution was diluted 100‐fold in the DPSC‐GM described above to a final concentration of 100 ng/mL.

### 2.4 Cell culture

**FIGURE 1:**
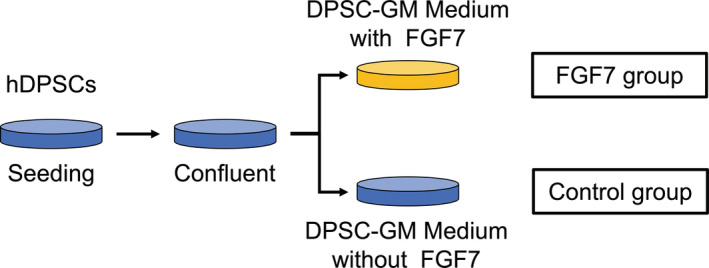
Schematic of the culture method of hDPSCs used for the *in vivo* study. hDPSCs treated with FGF7 are defined as the FGF7 group, while hDPSCs treated without FGF7 are defined as the control group

hDPSCs were seeded in 100‐mm dishes at a cell density of 5000 cells/cm^2^ (275,000 cells/dish) and were cultured in DPSC‐GM that was changed every 3 days at a temperature of 37°C with 5% carbon dioxide. hDPSCs were detached using Trypsin/EDTA and were passaged. When hDPSCs reached confluence after 2 passages, the medium was changed to DPSC‐GM containing FGF7, and this group was designated as the FGF7 group (Figure 1). The control group of DPSCs continued to use DPSC‐GM but without FGF7. The day when the culture medium was changed is defined as day 0.

**TABLE 1:** Primers used for quantitative reverse transcription polymerase chain reaction

Primer	Gene name	Assay ID
BMI1	BMI1 proto‐oncogene	Hs00409825_g1
AQP5	aquaporin 5	Hs00387048_m1
CK19	keratin 19	Hs00761767_s1
αSMA	smooth muscle alpha‐actin	Hs00426835_g1
VIMENTIN	vimentin	Hs00185584_m1
GAPDH (endogenous control)	glyceraldehyde‐3‐phosphate dehydrogenase	Hs02786624_g1

### 2.5 Proliferation assay of hDPSCs

The hDPSCs were seeded in 24 well dishes at a cell density of 5000 cells/cm^2^ (10,000 cells/well) and were collected at days 1, 3, 5, 7, 9, 11 and 13 in each group using a cell scraper. The hDPSCs were counted using a Coulter Counter (Beckman Coulter, California) and the number of cells was plotted.

### 2.6 Quantitative reverse transcription polymerase chain reaction (qRT‐PCR)

hDPSCs at days 3, 7 and 14 in each group were collected using a cell scraper. Total RNAs were extracted using a RNeasy Mini Kit (QIAGEN, Hilden, Germany) and were reverse transcribed into complementary DNAs (cDNAs) using ReverTra Ace qPCR RT Master Mix with gDNA Remover (TOYOBO, Osaka, Japan). qRT‐PCR was performed using TaqMan Gene Expression Assays (Applied Biosystems, Massachusetts). The target genes characterized in this study were: BMI1 (*BMI1*: Hs00409825_g1) as an undifferentiated marker, aquaporin 5 (*AQP5*: Hs00387048_m1) as an acinar cell marker, cytokeratin 19 (*CK19*: Hs00761767_s1) as a ductal epithelial cell marker, α‐smooth muscle actin (*αSMA*: Hs00426835_g1) as a myoepithelial cell marker, vimentin (*VIMENTIN*: Hs00185584_m1) as a fibroblast cell marker and glyceraldehyde‐3‐phosphate dehydrogenase (*GAPDH*: Hs02786624_g1) as an endogenous control (Table 1). qRT‐PCR was performed using a 7500 Fast Real‐Time PCR System (Applied Biosystems, Massachusetts, USA). mRNA expression levels were corrected based on *GAPDH* mRNA expression levels, and target gene expression levels were subjected to relative quantitative analysis.

### 2.7 Animals

This study was conducted in compliance with the Guidelines for the Treatment of Experimental Animals at the Tokyo Dental College (Approval Number: 193401). Adult male Sprague‐Dawley rats, each weighing approximately 200 g (Sankyo Lab Service, Tokyo, Japan), were used in this study. No animals suffered infection or death during the experimental period.

### 2.8 Preparation of collagen gels

An acid‐soluble type I collagen solution derived from porcine tendons was prepared to a concentration of 3.0 mg/mL at a pH of 3.0 (Cellmatrix Type I‐A; Nitta Gelatin, Osaka, Japan) for use in this study. This solution was quickly reconstituted into collagen gels with consistently high strength and transparency required for use.

### 2.9 Preparation of hDPSCs in collagen gels

hDPSCs cultured with or without FGF7 for 14 days were used in the *in vivo* study. Sheets of hDPSCs were collected using a cell scraper and were separated into single cells by pipetting. The supernatant was removed after centrifugation and a collagen gel solution at a concentration of 100,000 cells/50 μg was added to the remaining hDPSCs. The collagen gel solution containing hDPSCs, which was in a liquid condition, was reconstituted by incubation for 30 min in an incubator at 37°C to produce a gel and was used as a graft material.

### 2.10 Wounded rat SMGs and transplantation of hDPSCs

**FIGURE 2:**
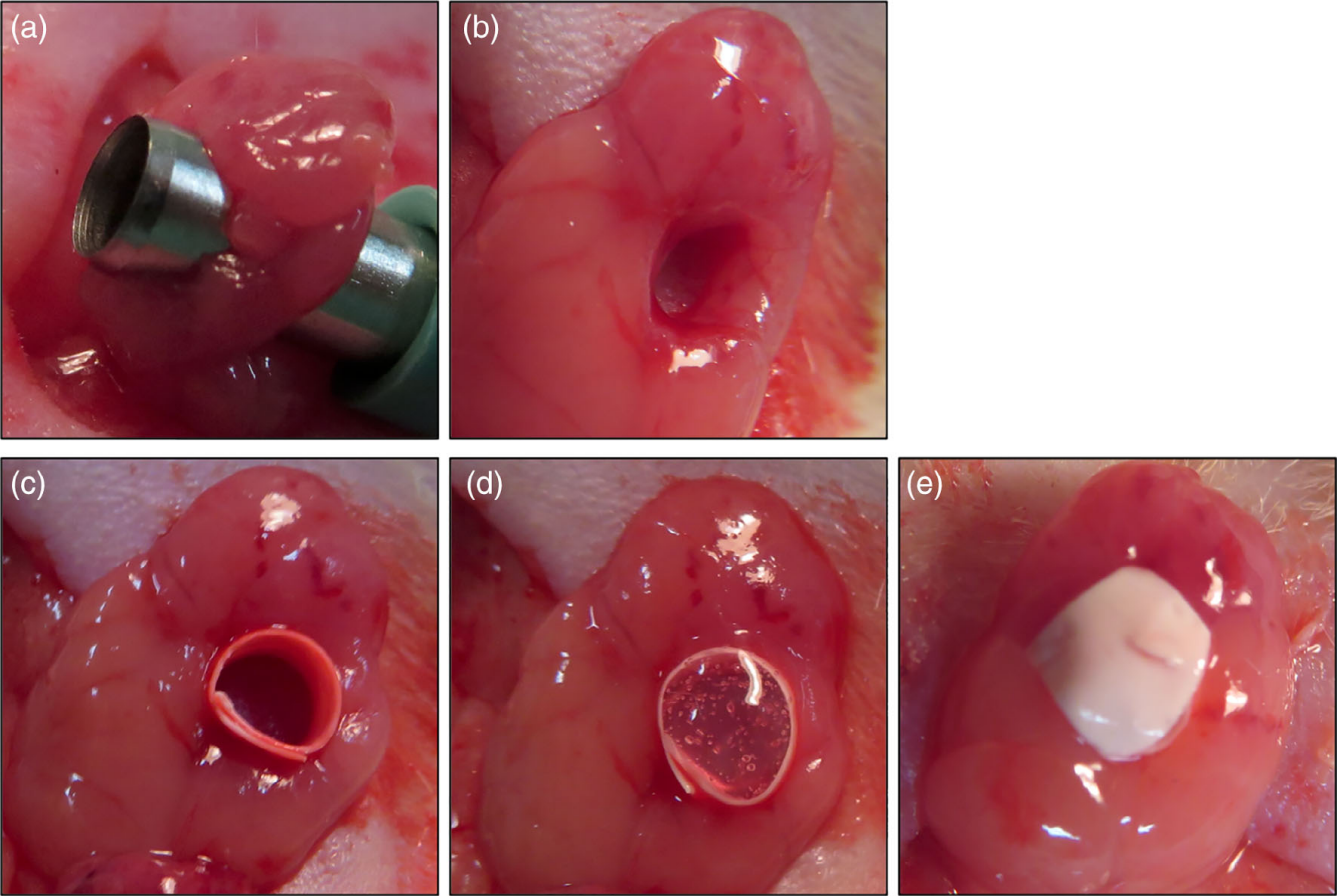
Method for the surgically wounded model of rat SMGs used for the *in vivo* study. A defect was created in each rat SMG using a 3‐mm diameter biopsy punch (a, b). A cylinder‐shaped Millipore membrane filter was inserted into each defect to prevent the invasion of surrounding cells and tissue (c). Type I collagen gel mixed with hDPSCs cultured for 14 days was transplanted inside the Millipore cylinder in the defect area (d). The collagen gel was covered with a Millipore membrane filter on the top and bottom of the cylinder (e)

The *in vivo* experiment was performed according to the method of Kobayashi et al. (Kobayashi et al., 2016). Each rat was sedated with isoflurane and was then anesthetized by an intraperitoneal injection of three mixed anesthetics (0.5 mL/100 g). The front of the neck of each rat was shaved and an approximately 2 cm incision was made in the skin at the midline of the neck with a surgical knife. The salivary glands were exposed, and defects were created using a 3 mm biopsy punch (Kai Industries, Gifu, Japan) without damaging the main artery or the main salivary duct (Figure 2a,b). The wounded area was covered by a Millipore membrane filter (MF‐Millipore, 0.45 μm pore size, HAWP01300; Merck Millipore, Massachusetts) to avoid the invasion of cells from the remaining salivary gland tissue. Each Millipore membrane was cut in advance to an appropriate size, made into a cylindrical shape and inserted into the wounded area. After insertion, the membrane was carefully spread to the wound margin (Figure 2c). The wound area lined with a Millipore membrane filter was then filled with collagen gel mixed with hDPSCs treated with FGF7 (FGF7 group) or without FGF7 (Control group) using an injection syringe (Figure 2d). In addition, Millipore membrane filters were placed on the top and bottom of each wound to avoid the overflow of gels (Figure 2e). The skin was then sutured using 4‐0 nylon thread.

### 2.11 Histological observations

Three rats in each group were euthanized using an overdose of somnopentyl at days 3 and 7 after the surgery (*n* = 12). Whole rat SMGs were resected and were immersed in 10% neutral buffered formalin for 24 h at room temperature. Specimens were dehydrated in ethanol before being embedded in paraffin. Paraffin sections, 4 μm in thickness, were cut horizontally using a sliding microtome. Paraffin sections were stained with hematoxylin and eosin (H‐E) for light microscopy observations.

### 2.12 Immunohistochemical observations

Paraffin sections of specimens at days 3 and 7 after the surgery were used for immunohistochemical observations. The paraffin sections were deparaffinized with xylol, after which they were activated for antigen by soaking them in distilled water prepared by diluting an immunosaber (Nisshin EM Corporation, Tokyo, Japan) with an electric pot at 98°C for 45 min. Sections were immersed in methanol containing 0.3% aqueous hydrogen peroxide at room temperature to block endogenous peroxidase activity. The sections were blocked for 60 min with 10% goat serum at room temperature to reduce non‐specific binding. Sections were reacted with primary antibodies overnight at 4°C. The primary antibodies used were: BMI1 (ab14389, 1:100; abcam, Cambridge, UK) as an undifferentiated marker, AQP5 (ab78486, 1:100; abcam, Cambridge, UK) as an acinar cell marker, αSMA (ab32575, 1:200; abcam, Cambridge, UK) as a myoepithelial cell marker, PanCK (ab86734, 1:50; abcam, Cambridge, UK) as a ductal epithelial cell marker and VIMENTIN (ab92547, 1:200; abcam, Cambridge, UK) as a fibroblast marker. The sections were then reacted with MACH 2 Universal HRP Polymer Detection (BRR522G, BIOCARE MEDICAL, California) as a peroxidase‐conjugated secondary antibody for 30 min at room temperature. After that, the sections were stained with DAB, and nuclei were stained with hematoxylin. The sections were observed with a light microscope. The number of cells that stained positive for each antibody at each time period in each group were counted at five locations chosen from randomly drawn rectangles, the size of 350 × 245 μm^2^, in the central part of the wound area.

### 2.13 Statistical analysis

Quantitative data are presented as means ± standard deviation (SD) and were analyzed using two‐way ANOVA in GraphPad Prism7 (MDF Corporation, Tokyo, Japan). Differences with a *p* value <.05 are considered statistically significant.

## 3 RESULTS

### 3.1 Proliferation of hDPSCs

**FIGURE 3:**
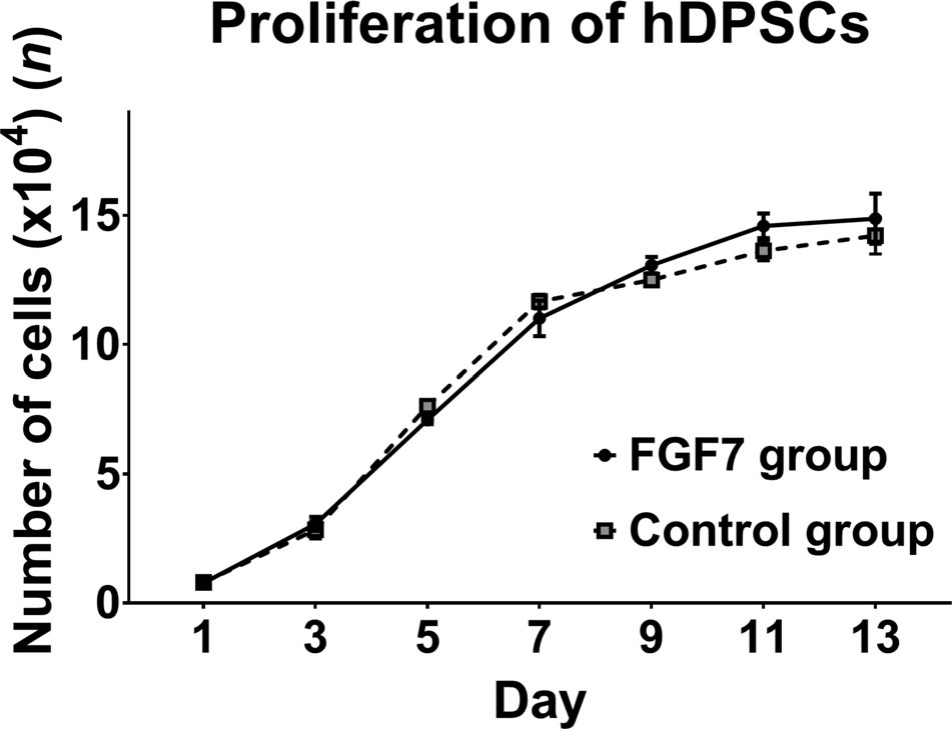
Proliferation of hDPSCs. There was no significant difference in proliferation *in vitro* between the FGF7 group and the control group from days 1 through 13

The proliferation of hDPSCs treated with or without FGF7 increased day by day until day 7, after which the cell numbers plateaued in both groups. There was no significant difference in proliferation between the FGF7 group and the control group (Figure 3).

### 3.2 qRT‐PCR analysis of hDPSCs

**FIGURE 4:**
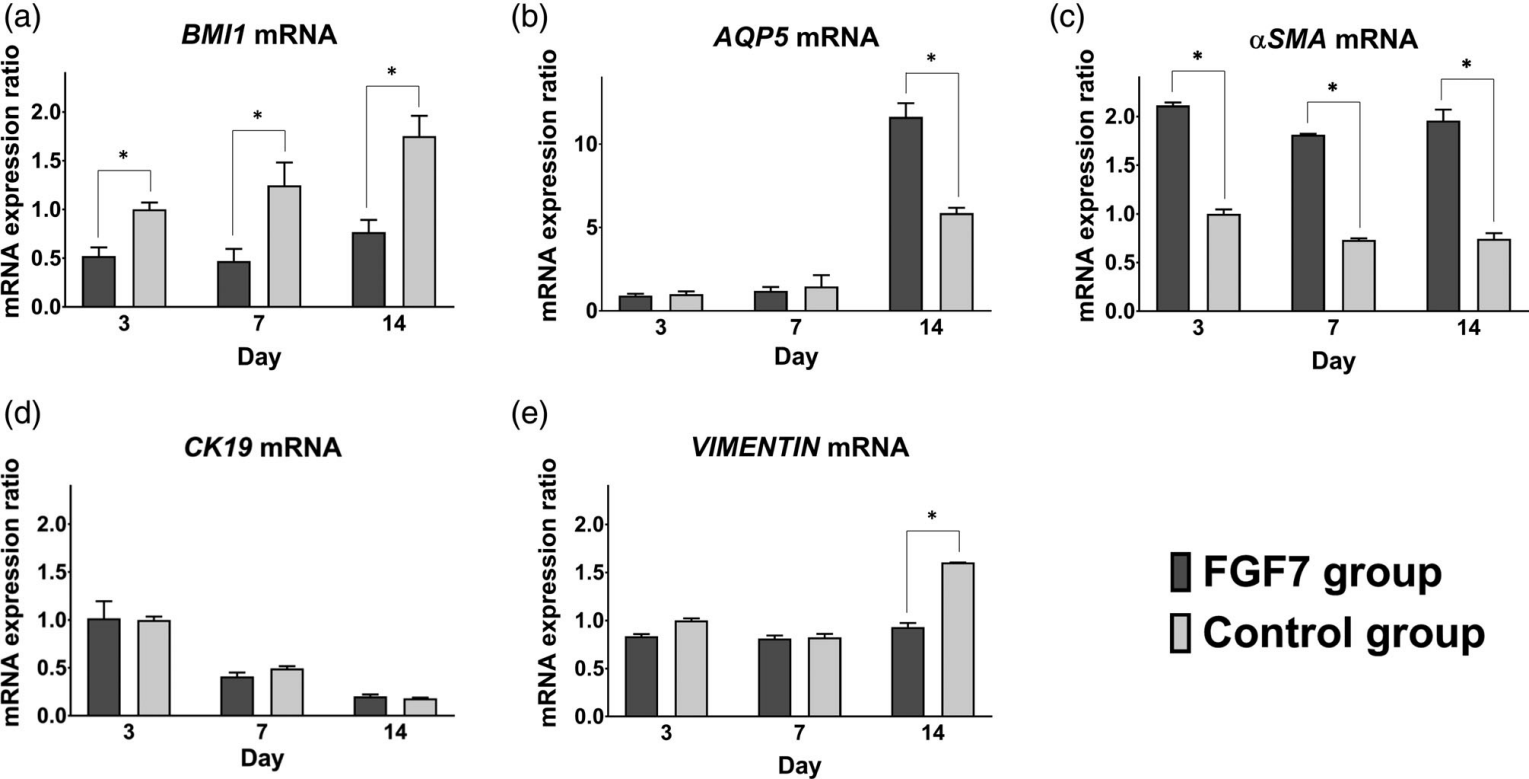
mRNA expression levels. qRT‐PCR analysis showed that the mRNA expression levels of cultured hDPSCs *in vitro* were as follows; *BMI1* was significantly lower at days 3, 7 and 14 (a), *AQP5* was significantly higher at day 14 (b), *αSMA* was significantly higher at days 3, 7 and 14 (c), *CK19* was not significantly different at days 3, 7 and 14 (d) in the FGF7 group compared to the control group. *VIMENTIN* was significantly lower at day 14 (e) in the FGF7 group compared to the control group. Bars: means ± standard deviation. Significance levels: *p < .05

The expression level of *BMI1* mRNA was significantly lower in the FGF7 group compared to the control group at days 3, 7 and 14 (Figure 4a). The expression level of *AQP5* mRNA was not significantly different at days 3 and 7, however it was significantly higher in the FGF7 group than in the control group at day 14 (Figure 4b). The expression level of *αSMA* mRNA was significantly higher in the FGF7 group compared to the control group at days 3, 7 and 14 (Figure 4c). The expression level of *CK19* mRNA was not significantly different in those groups at days 3, 7 and 14 (Figure 4d). The expression level of *VIMENTIN* mRNA also did not change significantly at days 3 and 7 but was significantly lower in the FGF7 group compared to the control group at day 14 (Figure 4e).

### 3.3 Histological observations

**FIGURE 5:**
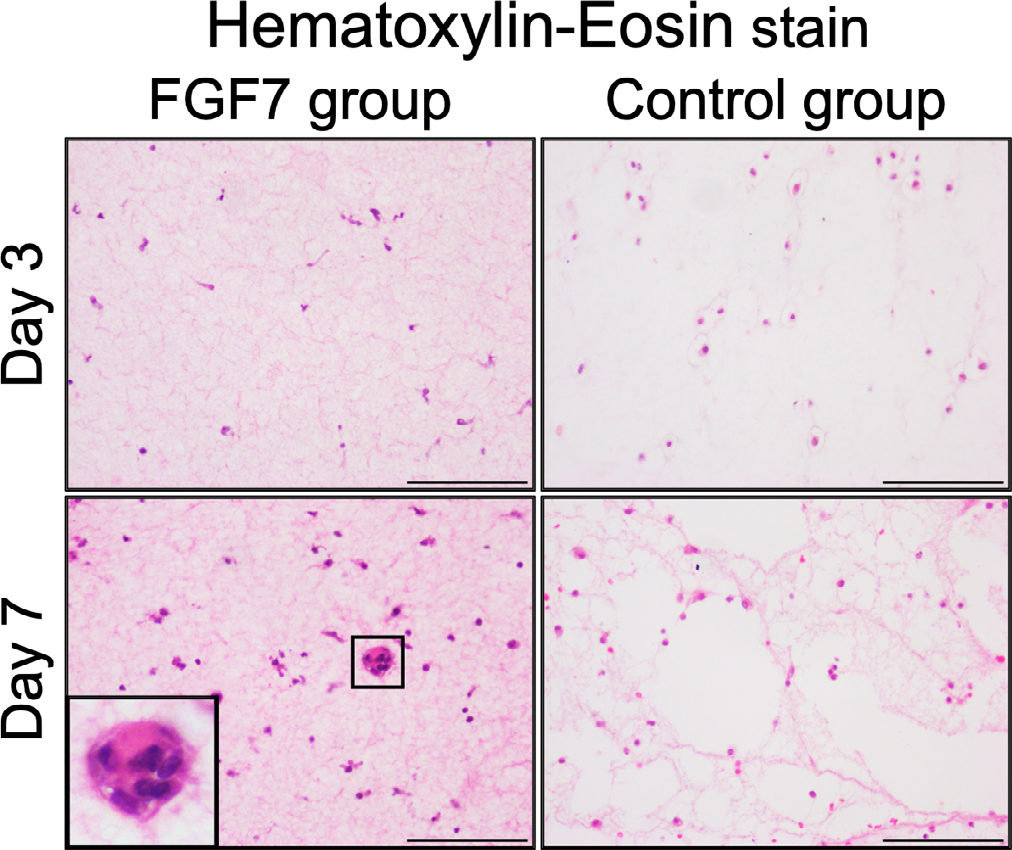
Hematoxylin–eosin stain. Wounded areas in rat SMGs assessed by hematoxylin–eosin staining in the *in vivo* study. hDPSCs proliferated in collagen gels in both groups, and the number of cells increased with time. Aggregations of cells were observed at day 7 in the FGF7 group. Original magnification; ×400. Scale bars: 100 μm

The hDPSCs proliferated in collagen gels in both the FGF7 and the control groups over time (Figure 5). Aggregations of cells and small island structures were sometimes observed at day 7 in the FGF7 group but were never seen in the control group.

### 3.4 Immunohistochemical observations

**FIGURE 6:**
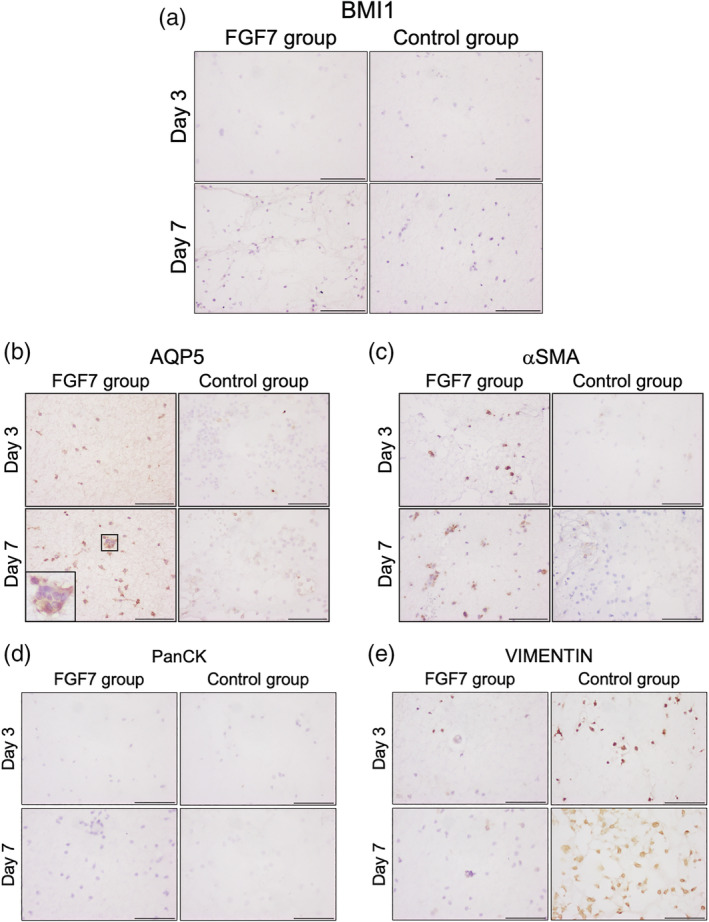
(a) Immunohistochemical staining of BMI1. Wounded area in rat SMGs assessed by immunohistochemical staining in the *in vivo* study. At days 3 and 7, few BMI1‐positive cells were observed in both groups. Original magnification: ×400; Scale bars: 100 μm. (b) Immunohistochemical staining of AQP5. Wounded area in rat SMGs assessed by immunohistochemical staining in the *in vivo* study. At days 3 and 7, more AQP5‐positive cells were observed in the FGF7 group. The aggregations of cells were AQP5‐positive. Original magnification: ×400; Scale bars: 100 μm. (c) Immunohistochemical staining of αSMA. Wounded area in rat SMGs assessed by immunohistochemical staining in the *in vivo* study. At days 3 and 7, more αSMA‐positive cells were observed in the FGF7 group. Original magnification: ×400; Scale bars: 100 μm. (d) Immunohistochemical staining of PanCK. Wounded area in rat SMGs assessed by immunohistochemical staining in the *in vivo* study. At days 3 and 7, few PanCK‐positive cells were observed in both groups (a, d). Original magnification: ×400; Scale bars: 100 μm. (e) Immunohistochemical staining of VIMENTIN. Wounded area in rat SMGs assessed by immunohistochemical staining in the *in vivo* study. At days 3 and 7, fewer VIMENTIN‐positive cells were observed in the FGF7 group. Original magnification: ×400; Scale bars: 100 μm

**FIGURE 7:**
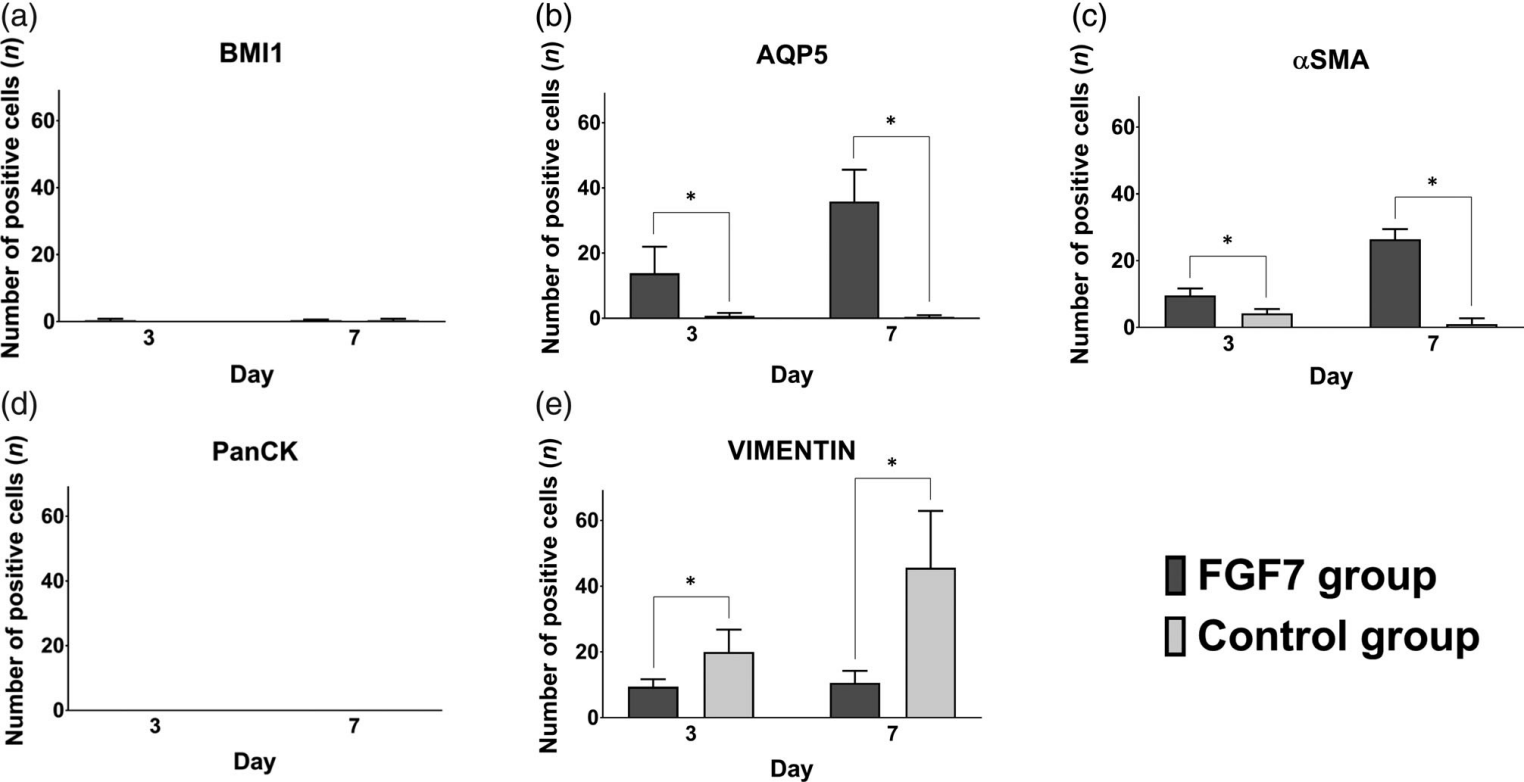
The number of immunopositive cells. Immunohistochemical staining showed that the numbers of immunohistochemically positive cells of hDPSCs in the *in vitro* study were as follows; at days 3 and 7, few BMI1‐ and PanCK‐positive cells were observed in both groups (a, d), the numbers of AQP5‐ and αSMA‐positive cells were significantly higher in the FGF7 group compared to the control group (b, c), the number of VIMENTIN‐positive cells was significantly lower in the FGF7 group compared to the control group (e). Bars: means ± standard deviation. Significance levels: *p < .05

Few BMI1‐positive cells were observed in hDPSCs treated with or without FGF7 at days 3 and 7 (Figure 6a), and there was no significant change in the number of BMI1‐positive cells between days 3 and 7 in both groups (Figure 7a). Many AQP5‐positive cells were observed at days 3 and 7 in the FGF7 group. But few AQP5‐positive cells were observed in the control group at either time (Figure 6b). The number of APQ5‐positive cells increased significantly at days 3 and 7 in the FGF7 group, but there was no significant difference in the control group at days 3 and 7 (Figure 7b). Aggregations of cells and small island structures were AQP5‐positive in the FGF7 group (Figure 6b). αSMA‐positive cells were observed at days 3 and 7 in the FGF7 group, and a few αSMA‐positive cells were observed in the control group at those times (Figure 6c). The number of αSMA‐positive cells increased significantly at days 3 and 7 in the FGF7 group, but there was no significant difference in the control group at those times (Figure 7c). PanCK‐positive cells were not observed in either group at days 3 or 7 (Figures 6d and 7d). VIMENTIN‐positive cells were observed in both groups at days 3 and 7 (Figure 6e). The number of VIMENTIN‐positive cells was significantly increased in the control group at days 3 and 7 compared with the FGF7 group, but there was no significant difference in the FGF7 group at days 3 and 7 (Figure 7e).

## 4 DISCUSSION

Generally, when salivary gland tissues are damaged and atrophy, degenerate, undergo necrosis and lose their original functions, non‐specific fibroblasts usually migrate into the damaged area and eventually scar tissue is formed. Only in cases when tissue stem cells migrate into the damaged area, incomplete regeneration might occur. Thus, it is important to avoid the migration of non‐specific fibroblasts from the surrounding tissue. To avoid this influx of contaminating fibroblasts, barrier membranes, such as Gore‐tex membranes, have been used to cover the defect areas. This barrier membrane technique is usually used in periodontal surgery and is called guided tissue regeneration (GTR) (Nyman, Lindhe, Karring, & Rylander, 1982). In this study, Millipore membrane filters that have a proper pore size and readily transport tissue fluids, including growth factors, were used as a barrier membrane instead of Gore‐tex membranes, which are waterproof and do not transport some types of molecules. This prevents cell migration but allows the transport of many kinds of growth factors from the wounded remaining salivary glands of the experimentally created defects. The results of this study showed that no capillaries or elongations of duct structures were observed and that transplanted hDPSCs could proliferate in the defect areas. These results suggest that this method is useful to investigate the transplanted hDPSCs treated with growth factors from the remaining salivary gland tissue.

In this study, the effects of hDPSCs treated with FGF7 on wound healing of rat SMGs were evaluated *in vitro* and *in vivo*. FGF7 is a member of the FGF family, which plays important roles in regulating the proliferation and differentiation of a wide range of cells (Morita & Nogawa, 1999). Currently, 22 distinct FGF proteins have been identified in humans, all of which are known as signaling molecules with structural similarities (Blaber, Disalvo, & Thomas, 1996; Ornitz & Itoh, 2001). It is known that FGF1 to FGF10 bind to FGF receptors on the membranes of fibroblasts and regulate their proliferation. Further, FGF7 also binds to the keratinocyte growth factor receptor on the membranes of some types of epithelial cells and regulates their proliferation. It has been reported that FGF7 is activated when SMGs are developing, and it promoted the component of epithelial branching in an *in vitro* study (Kera et al., 2014).

BMI1 is known as an undifferentiated marker of cells derived from the neural crest. In our *in vitro study*, expression levels of *BMI1* mRNA decreased over time in the FGF7 group compared to the control group, and this indicates a decrease in the multipotential ability of stem cells. This is consistent with the fact that only a few BMI1‐positive cells were seen immunohistochemically in the *in vivo* study. Taken together, the results suggest that FGF7 promotes the differentiation but not the proliferation of hDPSCs. VIMENTIN is mainly expressed in mesenchymal cells and is used as a fibroblast marker. The *VIMENTIN* mRNA expression level increased with time and there was a significant increase in the control group at day 14 compared with the FGF7 group in the *in vitro* study. Moreover, VIMENTIN‐positive cells were significantly increased in the control group compared with the FGF7 group in the *in vivo* immunohistochemical study. These results suggest that hDPSCs were differentiated into fibroblasts in the control group, suggesting the possibility of wound healing by scar tissue.

Salivary glands are composed of three kinds of epithelial cells; acinar cells, ductal epithelial cells and myoepithelial cells. In terms of salivary gland regeneration, these three kinds of epithelial cells along with structural characteristics are required to evaluate the regeneration of salivary glands.

The results demonstrated that the expression levels of *αSMA* mRNA increased significantly in the FGF7 group compared to the control group at all time periods. Furthermore, αSMA‐positive cells were significantly increased in the FGF7 group compared with the control group at all time periods. αSMA is a cytoskeletal protein of smooth muscle cells and is a myoepithelial cell marker. This suggests that FGF7 causes hDPSCs to differentiate into αSMA‐positive cells, which could be myoepithelial cells.

In addition, *AQP5* mRNA expression levels increased more in the FGF7 group at day 14 than in the control group in the *in vitro* study. Furthermore, the number of AQP5‐positive cells increased in the FGF7 group compared with the control group in the *in vivo* study (Ma et al., 1999). AQPs are proteins with pores that are present in acinar cell membranes (Agre, 2006). Since AQP5 can selectively pass only water molecules, it is involved in regulating water uptake into cells (Gonen & Walz, 2006). There are currently 13 known types of AQPs, and AQP5 is found in salivary glands (Cotroneo, Proctor, Paterson, & Carpenter, 2008; Nielsen et al., 2002) and is used as a marker of acinar cells. This suggests that FGF7 affects the differentiation of hDPSCs not only to αSMA‐positive cells, but also to AQP5‐positive cells, which could be acinar cells.

There was no change in the expression level of *CK19* mRNA in the *in vitro* study. Furthermore, only a few PanCK‐positive cells were observed in the *in vivo* immunohistochemical study. CK19 and PanCK are markers of ductal epithelial cells. This suggests that FGF7 does not affect the differentiation of hDPSCs into PanCK‐positive cells, which could be ductal epithelial cells.

In the *in vivo* study, aggregations of cells and small cell islands, which are like acini‐like structures, were positive for AQP5 at day 7 in the FGF7 group. The origin of acinar cells are progenitor cells of ductal epithelial cells that differentiate at the embryonic stage. However, in this study, no ductal epithelial cells, which should be negative for CK19 and PanCK, could be found around the AQP5‐positive cell masses. Also, there were no myoepithelial cells with basement membranes around the AQP5‐positive small island masses. Kobayashi et al. (2019) used scaffolds containing a basement membrane component typified by Matrigel and found acinar cells that were observed structurally. In this study, collagen gels, which have no basement membrane components, were used instead of Matrigel. This suggests that the use of basement membrane components and some other growth factors which cause cells to differentiate into ductal cells are probably necessary for the structural regeneration of salivary glands.

## 5 CONCLUSION

These results suggest that hDPSCs treated with FGF7 differentiate into AQP5‐positive and αSMA‐positive cells, and form AQP5‐positive cell aggregates when transplanted into experimentally damaged rat SMGs.

## CONFLICT OF INTEREST STATEMENT

The authors declare that there is no conflict of interest.

## Data Availability

The data that support the findings of this study are available from the corresponding author upon reasonable request.
